# 以B细胞成熟抗原为基础的CAR-T细胞治疗对复发/难治性多发性骨髓瘤患者肝功能影响的临床观察

**DOI:** 10.3760/cma.j.issn.0253-2727.2023.10.007

**Published:** 2023-10

**Authors:** 乾 孙, 岳坤 祁, 昆明 齐, 志凌 闫, 海 程, 伟 陈, 锋 朱, 威 桑, 德鹏 李, 江 曹, 明 施, 振宇 李, 开林 徐

**Affiliations:** 徐州医科大学血液病研究所，徐州医科大学附属医院血液科，江苏省骨髓干细胞重点实验室，徐州 221002 Hematology Institute of Xuzhou Medical University, Hematology Department of The Affiliated Hospital of Xuzhou Medical University, Jiangsu Provincial Key Laboratory of Bone Marrow Stem Cells, Xuzhou 221002, China

**Keywords:** 嵌合抗原受体T细胞, 多发性骨髓瘤, 肝功能, Chimeric antigen receptor T-cell, Multiple myeloma, Liver function

## Abstract

**目的:**

观察以B细胞成熟抗原（BCMA）为基础的嵌合抗原受体T细胞（CAR-T）治疗对复发/难治性多发性骨髓瘤（RRMM）患者肝功能的影响。

**方法:**

纳入2019年6月1日至2023年2月28日输注抗BCMA CAR-T细胞和输注抗BCMA联合抗CD19 CAR-T细胞的92例RRMM患者，回顾性分析其临床资料，观察患者ALT、AST、总胆红素（TBIL）、直接胆红素（DBIL）等指标变化并分析其与细胞因子释放综合征（CRS）的关系。

**结果:**

纳入92例患者进行分析，单纯输注抗BCMA CAR-T细胞组41例（44.6％），输注抗BCMA联合抗CD19 CAR-T细胞组51例（55.4％）。31例（33.7％）患者输注CAR-T细胞后发生2级以上肝功能指标改变，其中1项指标改变20例（21.7％），2项指标改变5例（5.4％），3项以上指标改变者6例（6.5％）。ALT、AST高峰时间中位数分别为d 17、d 14，超过2级中位持续时间分别为5.0 d和3.5 d；TBIL和DBIL高峰时间分别为d 19、d 21，超过2级时间均为4.0 d。开始发生CRS的中位时间为d8，发热高峰的中位时间为d 9。发生CRS的患者ALT、AST、TBIL高于未发生CRS的患者（*P*值分别为0.011、0.002和0.015），CRS是ALT、TBIL的影响因素（*OR*＝19.668，95％*CI* 18.959～20.173，*P*＝0.001）。通过抗CRS治疗和保肝治疗，肝功能指标改变可以逆转，无患者死于肝功能异常。

**结论:**

在以BCMA为基础的CAR-T细胞治疗的RRMM中，CRS是导致肝功能指标改变的重要因素，CAR-T细胞输注后发生的肝功能指标改变呈一过性，治疗后可逆转。

## Abstract (en)

**Objective:**

To observe the characteristics of the evolution of liver indexes in patients with relapsed/refractory multiple myeloma（RRMM）treated with CAR-T-cells based on BCMA.

**Methods:**

Retrospective analysis was performed of patients with RRMM who received an infusion of anti-BCMA CAR-T-cells and anti-BCMA combined with anti-CD19 CAR-T-cells at our center between June 1, 2019, and February 28, 2023. Clinical data were collected to observe the characteristics of changes in liver indexes such as alanine aminotransferase（ALT）, aspartate aminotransferase（AST）, total bilirubin（TBIL）, and direct bilirubin（DBIL）in patients, and its relationship with cytokine-release syndrome（CRS）.

**Results:**

Ninety-two patients were included in the analysis, including 41 patients（44.6％）in the group receiving a single infusion of anti-BCMA CAR-T-cells, and 51 patients（55.4％）in the group receiving an infusion of anti-BCMA combined with anti-CD19 CAR-T-cells. After infusing CAR-T-cells, 31 patients（33.7％）experienced changes in liver indexes at or above grade 2, which included 20 patients（21.7％）with changes in one index, five patients（5.4％）with changes in two indexes, and six patients（6.5％）with changes in three or more indexes. The median time of peak values of ALT and AST were d17 and d14, respectively, and the median duration of exceeding grade 2 was 5.0 and 3.5 days, respectively. The median time of peak values of TBIL and DBIL was on d19 and d21, respectively, and the median duration of exceeding grade 2 was 4.0 days, respectively. The median time of onset of CRS was d8, and the peak time of fever was d9. The ALT, AST, and TBIL of patients with CRS were higher than those of patients without CRS（*P*＝0.011, 0.002, and 0.015, respectively）. CRS is an independent factor that affects ALT and TBIL levels（*OR*＝19.668, 95％ *CI* 18.959–20.173, *P*＝0.001）. The evolution of liver indexes can be reversed through anti-CRS and liver-protection treatments, and no patient died of liver injury.

**Conclusion:**

In BCMA-based CAR-T-cell therapy for RRMM, CRS is an important factor causing the evolution of liver indexes. The evolution of liver indexes after CAR-T-cell infusion is transient and reversible after treatment.

嵌合抗原受体T细胞（CAR-T细胞）免疫疗法治疗复发/难治性多发性骨髓瘤（RRMM）已经取得了令人瞩目的疗效[1]，但不良事件也不容忽视。大多数RRMM患者输注CAR-T细胞后出现细胞因子释放综合征（CRS），表现为发热、低血压、低氧血症等[2]，并可能引起脏器功能损害。目前，输注CAR-T细胞后肝功能异常的报道较少[3]–[4]。我们总结了本中心接受CAR-T细胞免疫治疗的RRMM患者资料，观察以B细胞成熟抗原（BCMA）为基础的CAR-T细胞治疗RRMM后肝功能指标的改变，并分析了发生肝功能指标改变的可能机制。

## 病例与方法

1. 病例资料：本研究为单中心回顾性队列研究，纳入徐州医科大学附属医院血液科2019年6月1日至2023年2月28日输注抗BCMA CAR-T细胞和输注抗BCMA联合抗CD19 CAR-T细胞的RRMM患者，所有患者均入组临床试验（ChiCTR-OIC-17011271、ChiCTR-OIC-17011272）。纳入标准：①符合国际骨髓瘤工作组（IMWG）专家共识（2016年版）中RRMM诊断标准[5]；②18～69岁；③Karnofsky功能状态评分≥50分；④预期寿命超过12周且无活动性感染；⑤入组前100 d内无自体造血干细胞移植史；⑥充分了解本研究且签署知情同意书，依从性较好，输注CAR-T细胞后继续住院观察，出院后定期复诊。本研究获得徐州医科大学附属医院临床试验伦理委员会批准（批件号：XYFY2017-KL014-01、XYFY2017-KL013-01）。收集患者年龄、性别、M蛋白类型、既往治疗经过、CAR-T靶点、输注CAR-T细胞日期，输注后的体温、血压、血氧饱和度等临床资料；收集预处理前至输注后60 d内以下五项肝功能指标：ALT、AST、ALB、总胆红素（TBIL）、直接胆红素（DBIL），同时收集铁蛋白和IL-6、IL-1β、IL-10、IFN-α、TNF-α、C反应蛋白（CRP）等细胞因子检测结果以及应用托珠单抗、糖皮质激素、保肝药物等治疗情况。

2. CAR-T治疗方案：预处理方案为氟达拉滨（30 mg·m^−2^·d^−1^，−5～−3 d）+环磷酰胺（750 mg/m^2^，−5 d），输注抗BCMA CAR-T细胞（1～2）×10^6^/kg或输注抗BCMA CAR-T细胞、抗CD19 CAR-T细胞各1×10^6^/kg。

3. 肝功能指标和CRS的评估：参考CTCAE5.0评估肝功能指标改变的级别[6]。符合以下条件之一定义为2级以上肝功能指标改变：ALT≥120 U/L、AST≥105 U/L、ALB<30 g/L、TBIL≥30 µmol/L或DBIL≥12 µmol/L。CRS的评估采用ASTCT诊断分级标准[7]。

4. 统计学处理：采用SPSS 26.0、GraphPad Prism 9进行统计学分析。偏态分布的计量资料用中位数（*Q*_1_，*Q*_3_）描述，组间比较采用Mann-Whitney *U*检验。分类变量的组间比较采用卡方检验或Fisher精确概率法。将细胞因子和肝功能指标取对数，采用Spearman相关性分析细胞因子和肝功能指标的相关性，应用单因素Logistic回归分析影响肝功能指标的因素。双侧*P*<0.05为差异有统计学意义。

## 结果

1. 临床特征：共纳入92例RRMM患者，其中男53例（57.6％），女39例（42.4％），中位年龄57（31～69）岁；输注CAR-T细胞前中位治疗线数为4（2，4）线。单纯输注抗BCMA CAR-T细胞41例（44.6％），输注抗BCMA联合抗CD19 CAR-T细胞51例（55.4％）；79例（85.9％）患者发生CRS，从输注CAR-T细胞到发生CRS的中位时间为8（5，10）d，10例（10.9％）患者发生3级以上CRS。共3例（3.3％）患者EB病毒（EBV）-DNA>1×10^3^拷贝数/ml，均为输注单靶点抗BCMA CAR-T细胞的患者，肝功能指标均在正常范围；1例（1.1％）巨细胞病毒（CMV）-DNA达4.36×10^3^拷贝数/ml，为输注抗BCMA联合抗CD19 CAR-T细胞患者，其TBIL达60.1 µmol/L，ALT达128 U/L，经保肝治疗后好转；无患者出现乙型肝炎病毒（HBV）再激活。抗BCMA CAR-T和抗BCMA联合抗CD19 CAR-T患者临床特征和肝功能指标异常的发生率差异无统计学意义（表1）。

**表1:** 抗BCMA CAR-T和抗BCMA联合抗CD19 CAR-T治疗的RRMM患者临床特征及肝功能指标异常发生率的比较

临床特征	单纯抗BCMA（41例）	抗BCMA联合抗CD19（51例）	统计量	*P*值
性别（例，男/女）	27/14	26/25	2.059	0.151
年龄[岁，*M*（*Q_1_*，*Q_3_*）]	57（52，64）	57（51，63）	0.964	0.335
M蛋白类型[例数（%）]			Fisher	0.598
IgG	19（20.6）	22（23.9）		
IgA	9（9.8）	10（10.9）		
IgD	2（2.2）	7（7.6）		
轻链型	11（12.0）	12（13.0）		
既往治疗线数[*M*（*Q_1_*，*Q_3_*）]	4.0（2.0，4.0）	3.0（3.0，4.0）	0.247	0.805
是否发生CRS[例数（%）]			Fisher	0.372
发生CRS	37（40.2）	42（45.7）		
未发生CRS	4（4.3）	9（9.8）		
2级以上肝功能指标改变[例数（%）]				
ALT≥120 U/L	6（6.5）	5（5.4）	Fisher	0.531
AST≥105 U/L	12（13.0）	12（13.0）	0.388	0.533
ALB<30 g/L	1（1.1）	0（0）	Fisher	0.446
TBIL≥30 µmol/L	4（4.3）	4（4.3）	Fisher	1.000
DBIL≥12 µmol/L	3（3.2）	3（3.2）	Fisher	1.000

注 BCMA：B细胞成熟抗原；CAR-T：嵌合抗原受体T细胞；RRMM：复发/难治性多发性骨髓瘤；CRS：细胞因子释放综合征；TBIL：总胆红素；DBIL：直接胆红素

2. 肝功能指标的动态变化：92例患者中，31例（33.7％）输注CAR-T细胞后发生2级以上肝功能指标改变，其中1项指标改变20例（21.7％），2项指标改变5例（5.4％），3项及以上指标改变者6例（6.5％）。7例（7.6％）患者输注当天肝功能指标发生改变：5例（5.4％）TBIL高于预处理前且超过正常上限1.5倍；2例（2.2％）DBIL高于预处理前，分别为12.5 µmol/L、20.8 µmol/L。ALT、AST高峰时间中位数分别为d 17、d 14，超过2级中位持续时间分别为5.0 d和3.5 d；TBIL和DBIL高峰时间分别为d 19、21，超过2级时间均为4.0 d。发热高峰的中位时间为d 9，早于肝功能指标达到高峰的时间。CRS组发生2级以上肝功能指标改变27例（34.2％），发生2级以上ALT和（或）TBIL改变共14例（17.7％）。2级以上肝功能指标动态变化如表2所示。

**表2:** 接受CAR-T治疗的RRMM患者2级以上肝功能指标改变［d，*M*（*Q*_1_，*Q*_3_）］

指标	例数	从输注细胞到改变至2级的时间	维持2级以上的时间	从输注细胞到恢复2级以下的时间
ALT	11	14.5（10.0，18.0）	5.0（2.0，13.0）	20.0（15.0，28.0）
AST	24	13.0（11.0，18.0）	3.5（3.0，5.0）	18.0（15.5，25.0）
ALB	1	7.0	21.0	28.0
TBIL	8	15.5（13.0，21.0）	4.0（2.5，9.5）	22.5（16.5，28.0）
DBIL	6	18.0（10.0，21.5）	4.0（2.5，12.5）	24.0（15.5，28.0）

注 CAR-T：嵌合抗原受体T细胞；RRMM：复发/难治性多发性骨髓瘤；TBIL：总胆红素；DBIL：直接胆红素

3. 肝功能指标的亚组分析：根据CAR-T细胞靶点、是否发生CRS进行分组，比较各项肝功能指标在亚组间的差异。如表3所示，单纯输注抗BCMA CAR-T细胞的患者和输注抗BCMA联合抗CD19 CAR-T细胞的患者肝功能指标差异均无统计学意义，而发生CRS的患者ALT、AST、TBIL高于未发生CRS的患者，差异有统计学意义。

**表3:** 不同CAR-T细胞靶点及是否发生CRS的RRMM患者肝功能指标的亚组分析［*M*（*Q*_1_，*Q*_3_）］

项目	CAR-T细胞靶点				CRS			
	单纯抗BCMA（41例）	抗BCMA联合抗CD19（51例）	*z*值	*P*值	发生CRS（79例）	未发生CRS（13例）	*z*值	*P*值
ALT（U/L）	54.0（22.0，110.5）	44.0（22.0，83.0）	0.931	0.352	54.5（26.0，108.3）	24.5（13.8，40.0）	2.534	0.011
AST（U/L）	76.0（32.0，159.0）	64.0（34.0，128.0）	0.314	0.753	77.0（35.0，163.0）	27.5（17.5，53.5）	3.030	0.002
ALB（g/L）	32.5（30.1，36.1）	33.7（28.8，36.4）	0.723	0.470	32.7（29.1，36.2）	34.5（31.7，36.5）	0.847	0.397
TBIL（µmol/L）	17.9（13.8，27.0）	17.6（10.5，23.4）	1.367	0.172	17.9（13.1，26.6）	10.9（8.6，18.3）	2.440	0.015
DBIL（µmol/L）	5.7（5.4，8.2）	5.2（3.3，9.4）	1.037	0.300	5.7（4.1，9.5）	3.9（3.3，6.3）	1.644	0.100

注 CAR-T：嵌合抗原受体T细胞；CRS：细胞因子释放综合征；RRMM：复发/难治性多发性骨髓瘤；BCMA：B细胞成熟抗原；TBIL：总胆红素；DBIL：直接胆红素

4. 影响肝功能指标改变的因素分析：将发生2级以上ALT和（或）TBIL改变作为因变量，将性别、年龄、既往治疗线数、CAR-T细胞靶点、是否发生CRS作为自变量纳入单因素Logistic回归分析，结果显示：CRS是ALT和（或）TBIL改变的影响因素（*OR*＝19.668，95％*CI* 18.959～20.173，*P*＝0.001），详见表4。

**表4:** 影响RRMM患者肝功能指标改变的因素分析

因素	*OR*	95％ *CI*	*P*值
男性	0.410	0.134～1.255	0.118
年龄	1.067	0.985～1.157	0.112
既往治疗线数	0.724	0.465～1.128	0.154
CAR-T细胞靶点为抗BCMA联合抗CD19	0.773	0.247～2.414	0.657
发生CRS	19.668	18.959～20.173	0.001

注 CRS：细胞因子释放综合征；CAR-T：嵌合抗原受体T细胞；RRMM：复发/难治性多发性骨髓瘤；BCMA：B细胞成熟抗原

5. 铁蛋白、细胞因子与肝功能指标的关系：将每例患者铁蛋白和细胞因子峰值与肝功能指标峰值（ALB 为低值）进行相关性分析，结果显示：IL-10和ALB呈负相关（*r*＝−0.439，*P*＝0.001），铁蛋白与ALT（*r*＝0.324，*P*＝0.002）、AST（*r*＝0.505，*P*<0.001）呈正相关。

6. 肝功能指标异常的处理：14例发生2级以上ALT和（或）TBIL改变的患者均接受了保肝治疗，应用保肝药物种类数为2（2，3）种，保肝治疗中位时间10（8，17）d。9例患者接受了托珠单抗和（或）糖皮质激素治疗，其中单纯应用托珠单抗2例、单纯糖皮质激素2例，5例联合应用托珠单抗和糖皮质激素；另外5例患者仅通过保肝等对症治疗后好转。从输注CAR-T细胞到ALT恢复到2级以下的中位天数为20.0 d，TBIL恢复到2级以下的中位天数为22.5 d，无患者死于肝功能损害。

## 讨论

CAR-T细胞免疫治疗可出现CRS、免疫效应细胞相关神经毒性综合征（ICANS）、骨髓抑制、B细胞免疫缺陷等并发症[3],[8]–[9]。对CRS、ICANS等不良反应已有很多报道，但输注CAR-T细胞后出现肝功能异常的报道较少。

通常在输注CAR-T细胞前应用环磷酰胺、氟达拉滨清除淋巴细胞[10]。环磷酰胺是常用的烷化剂，通过肝脏代谢，单次750 mg/m^2^的剂量就可能导致肝功能指标改变[11]。氟达拉滨的代谢产物主要通过肾脏排泄，其对肝功能的直接损害作用较弱[12]。我们观察到5例（5.4％）患者d 0的TBIL高于预处理前且超过正常上限1.5倍，这提示预处理化疗是肝功能指标改变的原因之一。

清除淋巴细胞后发生的病毒感染可能导致肝功能异常，这些病毒包括嗜肝病毒和非嗜肝病毒，前者主要有HBV、丙型肝炎病毒（HCV），后者主要包括EB病毒（EBV）、巨细胞病毒（CMV）[13]–[15]。本中心研究发现，既往感染HBV的患者接受CAR-T细胞免疫治疗后发生HBV再激活风险很低[16]，本组病例没有发生HBV再激活。EBV或CMV感染均可能造成肝功能指标改变，我们观察到3例患者EBV滴度增高，1例CMV滴度增高，CMV滴度增高的患者TBIL达60.1 µmol/L，ALT达128 U/L，经过抗病毒治疗和保肝治疗后好转。

脱靶效应（on-target/off-tumor effect）也可以是导致器官损害的原因之一，肝细胞表达CD19和BCMA的水平很低，所以脱靶效应不是本研究中肝功能指标改变的主要原因[17]。但CAR-T细胞与肝细胞或胆管上皮细胞是否存在相互作用、表达CAR的细胞毒性T细胞（CTL）是否可能直接损伤肝细胞尚不明确。

CRS与单核/巨噬系统过度活化、炎症因子风暴、内皮细胞活化等因素有关。目前关于CRS与肝功能损伤关系的研究不多，Wei等[18]基于CAR-T治疗B细胞非霍奇金淋巴瘤（B-NHL）提出了CRS模型，该模型认为在CRS的特定阶段，扩增后系统性重新分布的活化CAR-T细胞可造成靶器官（如肝脏）损伤。我们发现，发热高峰在d 9，开始发生CRS的中位时间是d 8，而ALT、TBIL升高达2级的中位时间晚于CRS发生的时间（分别为d 14.5和d 15.5）。与B-NHL相似的是，MM发生CRS同样涉及CAR-T在骨髓微环境的扩增及后续再分布，因此我们推测肝损伤由CAR-T细胞在全身各系统的重新分布及肝脏单核-巨噬细胞系统的活化引起。但是，受限于研究疾病不同的病理生理特点，具体机制需要进一步探索。在发生CRS的患者中，14例（17.7％）发生2级以上ALT、TBIL改变，与文献报道大致相当[3],[19]；发生CRS患者AST、ALT、TBIL高于未发生CRS的患者，肝功能指标与IL-10、铁蛋白存在一定的相关性。因此，我们认为CRS是肝功能指标改变的主要原因[18]。

我们发现肝功能指标变化以转氨酶改变为主，其发生率高于胆红素改变。对ALT和（或）TBIL改变超过2级的患者，特别是同时存在CRS的患者，通过有效抗CRS治疗和积极保肝治疗可以较快逆转肝功能指标改变，本研究没有患者死于肝功能异常。

本研究回顾了以BCMA为基础的CAR-T细胞治疗RRMM的病例，发现CRS是导致肝功能指标改变的重要因素，CAR-T细胞治疗后发生肝功能指标改变呈一过性，过程可逆。
